# Supplementary material for: Modeling glioblastoma heterogeneity as a dynamic network of cell states
Source: Mol Syst Biol. 2021 Sep 16;17(9):e10105. doi: 10.15252/msb.202010105 (PMC8444284; doi:10.15252/msb.202010105)
Supplement: Supplementary file 5 — Source Data for Figure 3 [file MSB-17-e10105-s001.zip › Figure3A_sourcedata/GSEA_3065/nosigdb_state6.GseaPreranked.1622718443993/gsea_report_for_na_neg_1622718443993.html]

Report for na\_neg 1622718443993 [GSEA]

| GS  follow link to MSigDB | GS DETAILS | SIZE | ES | NES | NOM p-val | FDR q-val | FWER p-val | RANK AT MAX | LEADING EDGE || 1 | LARSSON\_STATE\_2 | Details ... | 85 | -0.87 | -3.40 | 0.000 | 0.000 | 0.000 | 650 | tags=80%, list=7%, signal=86% |
| 2 | LARSSON\_STATE\_3 | Details ... | 86 | -0.84 | -3.24 | 0.000 | 0.000 | 0.000 | 645 | tags=71%, list=7%, signal=76% |
| 3 | DOROSZKO\_U3180MG\_CLUSTER\_2 | Details ... | 463 | -0.61 | -2.90 | 0.000 | 0.000 | 0.000 | 1505 | tags=45%, list=17%, signal=52% |
| 4 | FELDMAN\_2020\_G1 | Details ... | 148 | -0.61 | -2.59 | 0.000 | 0.000 | 0.000 | 1842 | tags=57%, list=21%, signal=71% |
| 5 | VANGRONINGEN\_MES | Details ... | 367 | -0.54 | -2.55 | 0.000 | 0.000 | 0.000 | 1522 | tags=41%, list=17%, signal=48% |
| 6 | RICHARDS\_ZHONG\_NATURE\_2018\_NPCS\_UPREG | Details ... | 123 | -0.60 | -2.46 | 0.000 | 0.000 | 0.000 | 1778 | tags=54%, list=20%, signal=66% |
| 7 | SEURAT\_CELLCYCLE | Details ... | 88 | -0.63 | -2.46 | 0.000 | 0.000 | 0.000 | 1776 | tags=53%, list=20%, signal=66% |
| 8 | DOROSZKO\_U3013MG\_CLUSTER\_2 | Details ... | 204 | -0.54 | -2.40 | 0.000 | 0.000 | 0.000 | 2125 | tags=54%, list=24%, signal=69% |
| 9 | NB\_KI\_CLUSTER5 | Details ... | 85 | -0.60 | -2.35 | 0.000 | 0.000 | 0.000 | 2033 | tags=59%, list=23%, signal=76% |
| 10 | IVY\_CTMVP | Details ... | 43 | -0.68 | -2.33 | 0.000 | 0.000 | 0.000 | 481 | tags=42%, list=5%, signal=44% |
| 11 | OLSEN\_SCPLIKE | Details ... | 77 | -0.58 | -2.24 | 0.000 | 0.000 | 0.000 | 658 | tags=36%, list=7%, signal=39% |
| 12 | LARSSON\_STATE\_5 | Details ... | 99 | -0.56 | -2.24 | 0.000 | 0.000 | 0.000 | 86 | tags=59%, list=1%, signal=58% |
| 13 | DOROSZKO\_U3013MG\_CLUSTER\_0 | Details ... | 26 | -0.72 | -2.23 | 0.000 | 0.000 | 0.000 | 806 | tags=50%, list=9%, signal=55% |
| 14 | SUVA\_G2M\_PMID\_31327527 | Details ... | 43 | -0.64 | -2.23 | 0.000 | 0.000 | 0.000 | 2114 | tags=58%, list=24%, signal=76% |
| 15 | RICHARDS\_NEFTEL\_CELL\_2019\_G2\_M | Details ... | 43 | -0.64 | -2.22 | 0.000 | 0.000 | 0.000 | 2114 | tags=58%, list=24%, signal=76% |
| 16 | PARADA\_T1\_UP\_26461091 | Details ... | 62 | -0.59 | -2.20 | 0.000 | 0.000 | 0.000 | 1232 | tags=44%, list=14%, signal=50% |
| 17 | LARSSON\_STATE\_4 | Details ... | 101 | -0.54 | -2.14 | 0.000 | 0.000 | 0.000 | 1774 | tags=50%, list=20%, signal=63% |
| 18 | ANASTASSIOU\_MULTICANCER\_INVASIVENESS\_PMID\_22208948 | Details ... | 26 | -0.69 | -2.11 | 0.000 | 0.000 | 0.000 | 1021 | tags=58%, list=12%, signal=65% |
| 19 | DOROSZKO\_PERIVASCULAR\_INVASION | Details ... | 33 | -0.64 | -2.07 | 0.000 | 0.000 | 0.001 | 624 | tags=45%, list=7%, signal=49% |
| 20 | OLSEN\_MESENCHYMAL | Details ... | 69 | -0.55 | -2.04 | 0.000 | 0.000 | 0.002 | 704 | tags=39%, list=8%, signal=42% |
| 21 | DOROSZKO\_U3180MG\_CLUSTER\_3 |  | 467 | -0.42 | -2.02 | 0.000 | 0.000 | 0.006 | 2151 | tags=41%, list=24%, signal=51% |
| 22 | NB\_KI\_CLUSTER17 |  | 125 | -0.48 | -1.98 | 0.000 | 0.000 | 0.006 | 1497 | tags=38%, list=17%, signal=45% |
| 23 | FELDMAN\_2020\_M-EARLYG1 |  | 108 | -0.49 | -1.97 | 0.000 | 0.000 | 0.006 | 1368 | tags=39%, list=16%, signal=45% |
| 24 | ALLEN\_114\_OPCL1\_6MYT1 |  | 15 | -0.73 | -1.96 | 0.000 | 0.000 | 0.009 | 280 | tags=40%, list=3%, signal=41% |
| 25 | RICHARDS\_NEFTEL\_CELL\_2019\_G1\_S |  | 26 | -0.64 | -1.96 | 0.003 | 0.000 | 0.009 | 1580 | tags=58%, list=18%, signal=70% |
| 26 | FELDMAN\_2020\_NEURAL G0 |  | 368 | -0.42 | -1.95 | 0.000 | 0.000 | 0.010 | 1307 | tags=35%, list=15%, signal=40% |
| 27 | SUVA\_G1S\_PMID\_31327527 |  | 26 | -0.64 | -1.92 | 0.000 | 0.001 | 0.016 | 1580 | tags=58%, list=18%, signal=70% |
| 28 | NB\_KI\_CLUSTER18 |  | 69 | -0.51 | -1.91 | 0.000 | 0.001 | 0.019 | 694 | tags=32%, list=8%, signal=34% |
| 29 | IVY\_CT |  | 143 | -0.45 | -1.90 | 0.000 | 0.001 | 0.022 | 1098 | tags=34%, list=12%, signal=38% |
| 30 | SUVA\_MES1\_PMID\_31327527 |  | 37 | -0.57 | -1.89 | 0.000 | 0.001 | 0.028 | 459 | tags=32%, list=5%, signal=34% |
| 31 | RICHARDS\_NEFTEL\_CELL\_2019\_MES1 |  | 37 | -0.57 | -1.87 | 0.000 | 0.001 | 0.038 | 459 | tags=32%, list=5%, signal=34% |
| 32 | NB\_KI\_CLUSTER14 |  | 207 | -0.42 | -1.86 | 0.000 | 0.002 | 0.040 | 1869 | tags=36%, list=21%, signal=44% |
| 33 | RICHARDS\_NOWAKOWSKI\_SCIENCE\_2017\_MGE\_IPC2\_UPREG |  | 475 | -0.39 | -1.84 | 0.000 | 0.002 | 0.054 | 2120 | tags=40%, list=24%, signal=50% |
| 34 | FELDMAN\_2020\_S-G2 |  | 326 | -0.40 | -1.84 | 0.000 | 0.002 | 0.054 | 1787 | tags=40%, list=20%, signal=48% |
| 35 | DALMO\_BARCODENUMBER\_UNPUB |  | 190 | -0.42 | -1.83 | 0.000 | 0.002 | 0.058 | 1368 | tags=38%, list=16%, signal=45% |
| 36 | NB\_KI\_CLUSTER23 |  | 245 | -0.41 | -1.83 | 0.000 | 0.002 | 0.062 | 994 | tags=27%, list=11%, signal=30% |
| 37 | RICHARDS\_ZHONG\_NATURE\_2018\_ASTROCYTES\_UPREG |  | 224 | -0.40 | -1.81 | 0.000 | 0.002 | 0.076 | 1719 | tags=37%, list=20%, signal=45% |
| 38 | RICHARDS\_NOWAKOWSKI\_SCIENCE\_2017\_ASTROCYTE\_UPREG |  | 470 | -0.38 | -1.79 | 0.000 | 0.003 | 0.104 | 1318 | tags=26%, list=15%, signal=28% |
| 39 | FELDMAN\_2020\_LATEG1 |  | 91 | -0.45 | -1.78 | 0.000 | 0.003 | 0.109 | 1234 | tags=36%, list=14%, signal=42% |
| 40 | ALLEN\_111\_ASTROL1\_6FGFR3ETNPPL |  | 23 | -0.60 | -1.78 | 0.002 | 0.003 | 0.110 | 635 | tags=30%, list=7%, signal=33% |
| 41 | GAROFANO\_IAVARONE\_PPR\_SUBTYPE |  | 387 | -0.37 | -1.78 | 0.000 | 0.003 | 0.110 | 1935 | tags=34%, list=22%, signal=42% |
| 42 | ALLEN\_112\_ASTROL1FGFR3MT1G |  | 25 | -0.58 | -1.75 | 0.002 | 0.005 | 0.151 | 635 | tags=32%, list=7%, signal=34% |
| 43 | DOROSZKO\_U3054MG\_CLUSTER\_2 |  | 229 | -0.39 | -1.75 | 0.000 | 0.005 | 0.156 | 1764 | tags=36%, list=20%, signal=44% |
| 44 | FELDMAN\_2020\_G2-M |  | 284 | -0.38 | -1.75 | 0.000 | 0.005 | 0.163 | 1845 | tags=40%, list=21%, signal=49% |
| 45 | PARADA\_T2\_UP\_26461091 |  | 61 | -0.47 | -1.72 | 0.002 | 0.006 | 0.205 | 1880 | tags=51%, list=21%, signal=64% |
| 46 | RICHARDS\_NOWAKOWSKI\_SCIENCE\_2017\_VRG\_UPREG |  | 378 | -0.36 | -1.71 | 0.000 | 0.006 | 0.214 | 1998 | tags=35%, list=23%, signal=43% |
| 47 | ALMSTEDT\_RISK |  | 30 | -0.54 | -1.71 | 0.002 | 0.006 | 0.217 | 699 | tags=30%, list=8%, signal=32% |
| 48 | FELDMAN\_2020\_S |  | 150 | -0.40 | -1.70 | 0.000 | 0.007 | 0.249 | 1608 | tags=41%, list=18%, signal=50% |
| 49 | DEPRETER\_LOW\_NB\_RISK |  | 18 | -0.61 | -1.69 | 0.011 | 0.007 | 0.251 | 516 | tags=33%, list=6%, signal=35% |
| 50 | RICHARDS\_VERHAAK\_CANCERCELL\_2010\_MESENCHYMAL |  | 96 | -0.43 | -1.69 | 0.000 | 0.007 | 0.267 | 1273 | tags=24%, list=14%, signal=28% |
| 51 | ALLEN\_113\_ASTROL1FGFR3FOS |  | 24 | -0.56 | -1.68 | 0.003 | 0.007 | 0.281 | 635 | tags=33%, list=7%, signal=36% |
| 52 | RICHARDS\_VERHAAK\_CANCERCELL\_2010\_CLASSICAL |  | 111 | -0.40 | -1.66 | 0.000 | 0.009 | 0.334 | 2132 | tags=41%, list=24%, signal=53% |
| 53 | GANDAL\_IBD\_UP\_PMID29439242 |  | 54 | -0.46 | -1.64 | 0.012 | 0.011 | 0.394 | 2383 | tags=48%, list=27%, signal=66% |
| 54 | DOROSZKO\_U3054MG\_CLUSTER\_1 |  | 16 | -0.60 | -1.64 | 0.022 | 0.011 | 0.412 | 147 | tags=25%, list=2%, signal=25% |
| 55 | RICHARDS\_ZHONG\_NATURE\_2018\_OPC\_UPREG |  | 70 | -0.43 | -1.63 | 0.003 | 0.011 | 0.418 | 624 | tags=23%, list=7%, signal=24% |
| 56 | RICHARDS\_NEFTEL\_CELL\_2019\_AC |  | 32 | -0.50 | -1.63 | 0.007 | 0.011 | 0.424 | 694 | tags=34%, list=8%, signal=37% |
| 57 | SUVA\_AC\_PMID\_31327527 |  | 32 | -0.50 | -1.61 | 0.023 | 0.014 | 0.499 | 694 | tags=34%, list=8%, signal=37% |
| 58 | SEGERMAN\_MULTITHERAPY\_RESISTANCE\_FIG5A\_PMID\_27974212 |  | 22 | -0.55 | -1.61 | 0.019 | 0.014 | 0.500 | 1963 | tags=45%, list=22%, signal=58% |
| 59 | GANDAL\_AUTISM\_UP\_PMID29439242 |  | 80 | -0.42 | -1.59 | 0.005 | 0.015 | 0.541 | 406 | tags=21%, list=5%, signal=22% |
| 60 | RICHARDS\_NOWAKOWSKI\_SCIENCE\_2017\_OPC\_UPREG |  | 311 | -0.34 | -1.58 | 0.001 | 0.017 | 0.593 | 1486 | tags=27%, list=17%, signal=31% |
| 61 | DOROSZKO\_U3180MG\_CLUSTER\_5 |  | 31 | -0.49 | -1.57 | 0.014 | 0.017 | 0.603 | 1232 | tags=39%, list=14%, signal=45% |
| 62 | RICHARDS\_LIDDELOW\_NATURE\_2017\_A1\_REACTIVE\_ASTROCYTES |  | 19 | -0.54 | -1.56 | 0.032 | 0.018 | 0.637 | 1963 | tags=47%, list=22%, signal=61% |
| 63 | LARSSON\_STATE\_1 |  | 97 | -0.39 | -1.56 | 0.006 | 0.018 | 0.642 | 642 | tags=22%, list=7%, signal=23% |
| 64 | RICHARDS\_LIDDELOW\_NATURE\_2017\_A2\_REACTIVE\_ASTROCYTES |  | 28 | -0.50 | -1.55 | 0.021 | 0.020 | 0.676 | 479 | tags=32%, list=5%, signal=34% |
| 65 | KLING\_GBM\_PHENO\_PMID\_27667176 |  | 138 | -0.37 | -1.53 | 0.002 | 0.022 | 0.721 | 1852 | tags=33%, list=21%, signal=42% |
| 66 | GAROFANO\_IAVARONE\_LIST\_OF\_NEURAL\_PROGENITOR\_MARKER\_GENES |  | 82 | -0.39 | -1.52 | 0.013 | 0.024 | 0.748 | 1020 | tags=28%, list=12%, signal=31% |
| 67 | NB\_KI\_CLUSTER8 |  | 303 | -0.33 | -1.52 | 0.000 | 0.025 | 0.771 | 1897 | tags=31%, list=22%, signal=38% |
| 68 | RICHARDS\_ZHONG\_NATURE\_2018\_MICROGLIA\_UPREG |  | 188 | -0.34 | -1.50 | 0.002 | 0.028 | 0.811 | 1771 | tags=30%, list=20%, signal=36% |
| 69 | DOROSZKO\_U3054MG\_CLUSTER\_0 |  | 55 | -0.41 | -1.49 | 0.026 | 0.031 | 0.842 | 640 | tags=24%, list=7%, signal=25% |
| 70 | DOROSZKO\_U3180MG\_CLUSTER\_1 |  | 15 | -0.55 | -1.48 | 0.045 | 0.032 | 0.862 | 18 | tags=13%, list=0%, signal=13% |
| 71 | POLLEN\_OUTERRADIALGLIA\_PMID\_26406371 |  | 48 | -0.41 | -1.45 | 0.032 | 0.040 | 0.915 | 1439 | tags=31%, list=16%, signal=37% |
| 72 | GANDAL\_ALCOHOLADDICTION\_DN\_PMID29439242 |  | 56 | -0.40 | -1.45 | 0.030 | 0.041 | 0.919 | 1786 | tags=34%, list=20%, signal=42% |
| 73 | RICHARDS\_NEFTEL\_CELL\_2019\_OPC |  | 36 | -0.44 | -1.45 | 0.034 | 0.041 | 0.925 | 475 | tags=25%, list=5%, signal=26% |
| 74 | OUTERRADIALGLIA |  | 48 | -0.41 | -1.45 | 0.045 | 0.040 | 0.926 | 1439 | tags=31%, list=16%, signal=37% |
| 75 | SUVA\_OPC\_PMID\_31327527 |  | 36 | -0.44 | -1.45 | 0.054 | 0.040 | 0.928 | 475 | tags=25%, list=5%, signal=26% |
| 76 | ANGIOGENESIS\_GO\_0001525 |  | 20 | -0.50 | -1.44 | 0.057 | 0.040 | 0.930 | 344 | tags=20%, list=4%, signal=21% |
| 77 | GANDAL\_IBD\_DN\_PMID29439242 |  | 19 | -0.49 | -1.40 | 0.106 | 0.058 | 0.980 | 1542 | tags=47%, list=18%, signal=57% |
| 78 | IVY\_CTPAN |  | 99 | -0.35 | -1.39 | 0.032 | 0.059 | 0.983 | 1285 | tags=29%, list=15%, signal=34% |
| 79 | WANG\_CLASSICAL\_PMID\_28697342 |  | 35 | -0.42 | -1.37 | 0.086 | 0.068 | 0.993 | 2104 | tags=43%, list=24%, signal=56% |
| 80 | DALMO\_EXTINCTION\_UNPUB |  | 23 | -0.46 | -1.36 | 0.096 | 0.075 | 0.995 | 928 | tags=26%, list=11%, signal=29% |
| 81 | RICHARDS\_NOWAKOWSKI\_SCIENCE\_2017\_IN\_CTX\_CGE2\_UPREG |  | 224 | -0.31 | -1.35 | 0.021 | 0.076 | 0.995 | 990 | tags=19%, list=11%, signal=21% |
| 82 | WANG\_MESENCHYMAL\_PMID\_28697342 |  | 23 | -0.46 | -1.35 | 0.096 | 0.078 | 0.995 | 928 | tags=26%, list=11%, signal=29% |
| 83 | ALMSTEDT\_11Q\_MSIGDB |  | 296 | -0.28 | -1.32 | 0.016 | 0.099 | 0.999 | 1704 | tags=25%, list=19%, signal=30% |
| 84 | RICHARDS\_NOWAKOWSKI\_SCIENCE\_2017\_MGE\_RG1\_UPREG |  | 309 | -0.28 | -1.31 | 0.021 | 0.104 | 0.999 | 799 | tags=16%, list=9%, signal=17% |
| 85 | KRONA\_ROSEN\_WHITE\_MATTER\_INVASION |  | 177 | -0.30 | -1.30 | 0.034 | 0.107 | 0.999 | 2252 | tags=33%, list=26%, signal=44% |
| 86 | DOROSZKO\_U3013MG\_CLUSTER\_1 |  | 36 | -0.39 | -1.29 | 0.128 | 0.110 | 0.999 | 1485 | tags=25%, list=17%, signal=30% |
| 87 | GANDAL\_SCHIZOPHRENIA\_UP\_PMID29439242 |  | 74 | -0.34 | -1.26 | 0.110 | 0.137 | 1.000 | 1354 | tags=26%, list=15%, signal=30% |
| 88 | DOROSZKO\_U3054MG\_CLUSTER\_5 |  | 94 | -0.31 | -1.23 | 0.126 | 0.168 | 1.000 | 1446 | tags=24%, list=16%, signal=29% |
| 89 | TISIG\_PMID\_32649888 |  | 33 | -0.36 | -1.17 | 0.240 | 0.240 | 1.000 | 635 | tags=18%, list=7%, signal=20% |
| 90 | ALLEN\_115\_OLIGOL4\_6MOBPCOL18A1 |  | 23 | -0.40 | -1.17 | 0.272 | 0.241 | 1.000 | 2252 | tags=43%, list=26%, signal=58% |
| 91 | SUVA\_MES2\_PMID\_31327527 |  | 44 | -0.33 | -1.16 | 0.240 | 0.247 | 1.000 | 1232 | tags=32%, list=14%, signal=37% |
| 92 | RICHARDS\_NEFTEL\_CELL\_2019\_MES2 |  | 44 | -0.33 | -1.15 | 0.272 | 0.262 | 1.000 | 1232 | tags=32%, list=14%, signal=37% |
| 93 | NB\_KI\_CLUSTER7 |  | 255 | -0.25 | -1.13 | 0.168 | 0.287 | 1.000 | 3083 | tags=39%, list=35%, signal=59% |
| 94 | SINGH\_SOX2\_DOWNREGULATED\_TRANSCRIPTS\_PMID\_28122245 |  | 84 | -0.29 | -1.12 | 0.270 | 0.307 | 1.000 | 1623 | tags=26%, list=18%, signal=32% |
| 95 | ALLEN\_116\_OLIGOL4\_6OPALIN |  | 20 | -0.37 | -1.07 | 0.330 | 0.402 | 1.000 | 2848 | tags=55%, list=32%, signal=81% |
| 96 | RICHARDS\_NOWAKOWSKI\_SCIENCE\_2017\_IN\_STR\_UPREG |  | 174 | -0.24 | -1.06 | 0.323 | 0.422 | 1.000 | 1127 | tags=17%, list=13%, signal=19% |
| 97 | DOROSZKO\_U3180MG\_CLUSTER\_4 |  | 162 | -0.25 | -1.05 | 0.342 | 0.431 | 1.000 | 1535 | tags=21%, list=17%, signal=25% |
| 98 | DOROSZKO\_U3054MG\_CLUSTER\_10 |  | 235 | -0.23 | -1.03 | 0.365 | 0.471 | 1.000 | 1820 | tags=24%, list=21%, signal=29% |
| 99 | DOROSZKO\_U3054MG\_CLUSTER\_6 |  | 111 | -0.25 | -1.01 | 0.429 | 0.515 | 1.000 | 2504 | tags=37%, list=28%, signal=51% |
| 100 | NB\_KI\_CLUSTER11 |  | 50 | -0.29 | -1.01 | 0.432 | 0.510 | 1.000 | 627 | tags=14%, list=7%, signal=15% |
| 101 | NB\_KI\_CLUSTER24 |  | 165 | -0.23 | -1.00 | 0.466 | 0.530 | 1.000 | 3332 | tags=42%, list=38%, signal=67% |
| 102 | ALMSTEDT\_ALK |  | 48 | -0.28 | -0.98 | 0.504 | 0.567 | 1.000 | 23 | tags=6%, list=0%, signal=6% |
| 103 | KRONA\_ROSEN\_PERIVASCULAR\_INVASION |  | 43 | -0.29 | -0.98 | 0.495 | 0.562 | 1.000 | 1994 | tags=35%, list=23%, signal=45% |
| 104 | NB\_KI\_CLUSTER6 |  | 463 | -0.20 | -0.98 | 0.543 | 0.570 | 1.000 | 1976 | tags=24%, list=22%, signal=30% |
| 105 | IVY\_LE |  | 25 | -0.32 | -0.95 | 0.523 | 0.629 | 1.000 | 1909 | tags=40%, list=22%, signal=51% |
| 106 | NB\_KI\_CLUSTER15 |  | 146 | -0.21 | -0.91 | 0.677 | 0.735 | 1.000 | 3117 | tags=36%, list=35%, signal=55% |
| 107 | NB\_KI\_CLUSTER20 |  | 16 | -0.33 | -0.89 | 0.610 | 0.767 | 1.000 | 2197 | tags=31%, list=25%, signal=42% |
| 108 | GANDAL\_BIPOLAR\_UP\_PMID29439242 |  | 58 | -0.24 | -0.87 | 0.707 | 0.797 | 1.000 | 962 | tags=16%, list=11%, signal=17% |
| 109 | RICHARDS\_CAHOY\_JNEUROSCI\_2011\_ASTRO\_MATURE |  | 473 | -0.18 | -0.86 | 0.908 | 0.807 | 1.000 | 2137 | tags=25%, list=24%, signal=31% |
| 110 | ALMSTEDT\_17Q\_MSIGDB |  | 340 | -0.18 | -0.86 | 0.847 | 0.812 | 1.000 | 1971 | tags=23%, list=22%, signal=28% |
| 111 | DOROSZKO\_U3054MG\_CLUSTER\_3 |  | 191 | -0.19 | -0.84 | 0.857 | 0.841 | 1.000 | 2524 | tags=32%, list=29%, signal=45% |
| 112 | GANDAL\_BIPOLAR\_DN\_PMID29439242 |  | 17 | -0.30 | -0.83 | 0.679 | 0.847 | 1.000 | 2279 | tags=47%, list=26%, signal=63% |
| 113 | GANDAL\_ALCOHOLADDICTION\_UP\_PMID29439242 |  | 134 | -0.20 | -0.82 | 0.868 | 0.868 | 1.000 | 911 | tags=12%, list=10%, signal=13% |
| 114 | PARADA\_T2\_DN\_26461091 |  | 38 | -0.24 | -0.81 | 0.776 | 0.874 | 1.000 | 1922 | tags=29%, list=22%, signal=37% |
| 115 | GANDAL\_DEPRESSION\_DN\_PMID29439242 |  | 31 | -0.21 | -0.68 | 0.920 | 0.979 | 1.000 | 2756 | tags=39%, list=31%, signal=56% |
| 116 | PARADA\_T1\_DN\_26461091 |  | 36 | -0.20 | -0.67 | 0.948 | 0.979 | 1.000 | 1683 | tags=22%, list=19%, signal=27% |
| 117 | NB\_KI\_CLUSTER9 |  | 19 | -0.19 | -0.54 | 0.981 | 0.995 | 1.000 | 1540 | tags=16%, list=18%, signal=19% |
Table: Gene sets enriched in phenotype **na **[plain text format]****
